# Comparing efficacy of different scoring models to predict hepatic encephalopathy after TIPS in cirrhotic patients

**DOI:** 10.1080/07853890.2025.2514082

**Published:** 2025-06-06

**Authors:** Xin-Jian Xu, Liang Yin, Yi-Jiang Zhu, Dong Lu, Xiang-Zhong Huang, Wei-Fu Lv, Chun-Ze Zhou, De-Lei Cheng

**Affiliations:** a Department of Interventional Radiology, Jiangyin Hospital Affiliated to Nantong University, Jiangyin, China; b Department of Interventional Radiology, The First Affiliated Hospital of USTC, Division of Life Sciences and Medicine, University of Science and Technology of China, Hefei, China

**Keywords:** TIPS, hepatic encephalopathy, Child-Pugh score, MELD score, CLIFC-AD score, FIPS score

## Abstract

**Background:**

Assessing hepatic encephalopathy (HE) risk post-transjugular intrahepatic portosystemic shunt (TIPS) in cirrhotic patients is crucial. This study compares the predictive performance of Child-Pugh and Model for End-Stage Liver Disease (MELD), CLIFC-AD and Freiburg index of post-TIPS survival (FIPS) scores for overt and severe HE. To compare the predictive value of Child-Pugh, MELD, CLIFC-AD and FIPS scores for overt and severe HE post-TIPS in cirrhotic patients.

**Materials and methods:**

We retrospectively analysed data from 406 cirrhotic TIPS patients (January 2017–January 2021). Scoring models were assessed for differentiation (C-index), calibration, clinical utility and overall performance at 1, 3, 6 and 12 months post-TIPS.

**Results:**

Predictive performance for overt HE post-TIPS was low across models. FIPS had superior predictive ability for severe HE at 1 and 12 months post-TIPS (C-index: 0.781, 0.705). FIPS and CLIFC-AD showed good predictive capacity for severe HE in sarcopenic patients at 1 and 12 months (FIPS: C-index 0.863, 0.757; CLIFC-AD: C-index 0.748, 0.732). FIPS had the highest hazard ratio for severe HE (HR = 3.520, 95% CI: 2.134–5.807) and CLIFC-AD for overt HE (HR = 2.132, 95% CI: 1.581–2.874).

**Conclusion:**

FIPS and CLIFC-AD scores demonstrate significant predictive ability for severe HE post-TIPS, particularly in sarcopenic patients.

## KEY MESSAGES

- The study compares Child-Pugh, MELD, CLIFC-AD and FIPS scores for predicting hepatic encephalopathy post-TIPS.
- FIPS excels in predicting post-TIPS severe hepatic encephalopathy.
- FIPS and CLIFC-AD scores effectively predict severe hepatic encephalopathy in sarcopenic patients.

## Introduction

Transjugular intrahepatic portosystemic shunt (TIPS) is a well-established therapeutic intervention for portal hypertension (PHT) in liver cirrhosis (LC) patients. It is instrumental in managing cirrhosis complications such as oesophageal variceal bleeding and refractory ascites. TIPS involves the creation of a conduit between the hepatic and portal veins through a minimally invasive [1,2] approach, typically reinforced with a polytetrafluoroethylene (PTFE)-covered stent to maintain patency. The Baveno VII consensus on PHT provides comprehensive guidelines for pre-emptive TIPS, recommending its performance within 72 h and ideally within 24 h, for oesophageal variceal bleeding – particularly in patients with type 1 or type 2 gastroesophageal varices. Criteria for pre-emptive TIPS include a Child-Pugh score below 14 in class C patients, a score above 7 in class B patients with active bleeding during endoscopy, or a hepatic venous pressure gradient over 20 mmHg at the time of bleeding (strong recommendation and high-quality evidence) [3]. For variceal bleeding unresponsive to pharmacological and endoscopic treatments, TIPS using PTFE-covered stents is the recommended salvage therapy (strong recommendation, moderate-quality evidence) [3]. This methodology is endorsed by international and Chinese clinical practice guidelines, particularly for refractory ascites [1] management, highlighting the procedure’s effectiveness based on the strength of the recommendation and the evidence supporting it.

Although TIPS is an effective treatment, it is associated with significant complications, especially hepatic encephalopathy (HE) [4]. HE is a neuropsychiatric syndrome stemming from liver failure and/or portal-systemic shunting, manifesting in a spectrum of cognitive disturbances ranging from mild to severe. These disturbances can escalate from subclinical alterations to coma. The West-Haven grading system classifies the severity of HE into five levels: minimal HE (grade 0) and grades I through IV [5]. Covert HE encompasses minimal HE and Grade I HE, while overt HE includes grades II through IV. The most acute forms, severe HE (grades III and IV) [6,7], require intensive hospital treatment to manage the symptoms effectively.

The incidence of overt HE after TIPS ranged from 25% to 45% [8]. HE reduces patients’ quality of life and escalates the mortality risk. HE adversely affects daily living, social interaction, alertness, emotional behaviour, physical activity, sleep quality, work performance, family management and leisure pursuits [9]. Furthermore, compared to grade 0 HE patients, the mortality risk is more pronounced in patients with Grade II HE post-TIPS, with a hazard ratio (HR) of 1.56. This risk escalated dramatically for patients with grade III-IV HE, evidenced by an HR of 3.68 [10]. A recent study has revealed that HE treatment costs have markedly increased over the last two decades [11]. Specifically, from 1994 to 2003, the total hospitalization costs for HE reached $5.9 billion; in 2003, these costs amounted to $1.3 billion [11]. An alarming rise is observed when comparing the figures from 2005, at $4.88 billion, to those in 2009, which soared to $7.25 billion [11].

Given the significant social and economic burden, addressing HE has become a primary concern in the long-term management of TIPS patients. Accurate prediction of HE risk and the identification of high-risk patients prior to the procedure are vital. To achieve this, clinicians can use different scoring models to pre-select suitable patients for TIPS, opt for smaller diameter dilation balloons during the procedure and ensure close monitoring post-procedure. Adopting these measures can facilitate the prevention and early treatment of post-TIPS HE. Additionally, these strategies can help conserve valuable medical resources and reduce the cost burden associated with HE.

A meta-analysis of 30 studies highlighted that preoperative liver function levels are an independent and predictive factor for HE after TIPS procedures [12]. Recent advancements in scoring models, notably the chronic liver failure consortium-acute decompensation (CLIFC-AD) score and the Freiburg index of post-TIPS survival (FIPS) score, have demonstrated superior prognostic discrimination compared to traditional metrics such as the Child-Pugh and Model for End-Stage Liver Disease (MELD) scores [13,14]. Despite these advancements, direct comparisons between traditional liver function scores (Child-Pugh and MELD) and newer models (CLIFC AD and FIPS) in forecasting HE post-TIPS remain unexplored. This lacuna motivated our investigation, which involved a clinical assessment of 406 patients who underwent TIPS with covered stents and were observed for over 12 months. Our study aimed to compare the effectiveness of the Child-Pugh, MELD, CLIF-C AD and FIPS scores in predicting overt and severe HE following TIPS. Furthermore, we examined the predictive capacity of these four scoring systems for overt and severe HE in patients with and without sarcopenia.

## Materials and methods

### Research design and data sources

This study presents a retrospective analysis of cirrhotic patients with PHT who underwent TIPS between January 2017 and January 2021 at the First Affiliated Hospital of the University of Science and Technology of China in Hefei, Anhui, People’s Republic of China. We leveraged an extensive prospective database, encompassing baseline clinical and laboratory characteristics, detailed records of the TIPS procedures and comprehensive follow-up evaluations. The use of this database was predicated on prior written informed consent obtained from all participating patients, negating the need for further consent for inclusion in this retrospective analysis. Our research complies with the TRIPOD (transparent reporting of a multivariable prediction model for Individual Prognosis or Diagnosis) reporting standards [15], ensuring transparency and thoroughness. Additionally, the study adheres to the ethical principles of the 1975 Declaration of Helsinki and has received approval from the Ethics Review Committee of the First Affiliated Hospital of USTC, ethical approval reference number (2023-RE-283).

Inclusion criteria for the study were: 1. Clinically diagnosed cirrhosis (histological examination consistent with the diagnosis of cirrhosis, clinical manifestations, laboratory tests and imaging features consistent with the diagnosis of cirrhosis); 2. Treatment with TIPS due to PHT-related complications such as gastrointestinal bleeding, refractory ascites, etc. Exclusion criteria: (1) Patients who had undergone TIPS before inclusion in the database; (2) advanced hepatocellular carcinoma (HCC) exceeding the Milan criteria for liver transplantation (single tumour diameter <5cm, or fewer than three nodules with each nodule’s maximum diameter <3cm); (3) other extrahepatic malignancies or acute severe diseases resulting in a life expectancy of less than 6 months; (4) no CT scan prior to TIPS (preoperative paravertebral lumbar muscle thickness could not be measured); (5) Death or loss to follow-up within 12 months post-TIPS. All patients were informed about the benefits, risks and complications associated with the TIPS procedure, and written informed consent was obtained.

### TIPS procedure

All TIPS procedures were conducted by a trio of skilled interventional radiologists, following the clinical practice guidelines for TIPS established by the Interventional Branch of the Chinese Medical Doctor Association [1]. The technique involved creating a conduit between the hepatic and portal veins through a puncture, followed by the insertion of either an 8 mm VIATORR self-expanding polytetrafluoroethylene-covered stent (W.L. Gore, Flagstaff, AZ) or a combination of an 8 mm covered stent (Bard E•LUMINEXX) with an 8 mm bare stent (Bard FLUENCY Plus). If required, coil and/or medical glue were employed for variceal embolization. The portal pressure gradient (PPG) was meticulously measured before and after the shunt creation to evaluate the procedure’s success. The objective was to lower the PPG to below 12 mmHg post-TIPS or to achieve a more than 50% reduction from the baseline PPG.

### Clinical follow-up, risk scoring, sarcopenia and endpoint events

#### Clinical follow-up

Patients were scheduled for outpatient follow-up after TIPS at 1, 3, 6 and 12 months post-procedure. Subsequently, annual follow-ups were conducted at outpatient clinics, complemented by quarterly telephone interviews. These follow-ups comprised laboratory tests, abdominal ultrasonography, contrast-enhanced CT and clinical evaluations for variceal bleeding, ascites, HE and survival status. In cases of shunt dysfunction, corrective measures involved balloon dilation of the existing stent or placement of a new stent in the original stent. The follow-up period continued until September 2023, or until liver transplantation or the patient’s demise, whichever came first.

#### Risk scoring

The study retrospectively estimated HE risk following TIPS using four database scoring models: Child-Pugh score, MELD score, CLIFC-AD score and FIPS score.

#### Sarcopenia

CT is recognized as the gold standard for evaluating sarcopenia in individuals with cirrhosis [16]. This study assessed sarcopenia retrospectively by measuring the transversal psoas muscle thickness (TPMT) using upper abdominal CT. Two experienced radiologists, each with over five years of experience and blinded to the patients’ clinical details and outcomes, independently conducted the measurements. The mean value of these measurements was subsequently used for statistical analysis. The CT images were sourced from the Picture Archiving and Communication System (PACS), and the psoas muscle thickness was measured utilizing tools provided by the American GE AW 4.7 workstation. Specifically, the TPMT was measured at the umbilical level on the right side, with TPMT being the maximal horizontal diameter perpendicular to the longest axis of the right psoas muscle. This measurement was normalized for patient height, yielding a ratio of TPMT to height (TPMT/H). Following the methodology of Paternostro R and colleagues [17], sarcopenia was defined as TPMT/*H* < 10.7 mm/m in males and TPMT/*H* < 7.8 mm/m in females. For the present study, patients were divided into a sarcopenic group (*n* = 148) and a non-sarcopenic group (*n* = 258).

#### Endpoint events

All scores were determined using laboratory results three days before the TIPS stents were placed. The West-Haven grading criteria [5] were utilized to diagnose and classify HE: covert HE includes minimal HE and Grade I HE, while Grades II-IV are considered overt HE. Furthermore, severe HE encompassed Grades III-IV, necessitating hospitalization for treatment [6,7]. The development of overt HE and severe HE served as the endpoint events for this study.

### Statistical analysis

The measurement data with normal distribution are presented as the mean and standard deviation (x ± s), and t-tests were employed for comparison. Conversely, the median and interquartile range [M(P25, P75)] were used for the data that did not follow a normal distribution, along with rank-sum tests for intergroup comparisons. Categorical variables were presented as the number of cases and percentages and analysed using chi-square tests or Fisher’s exact test when appropriate.

The predictive power of each scoring outcome was evaluated by determining HR using the Cox regression model. Additionally, R^2^ and Brier scores were calculated. Further analysis employed time-dependent ROC curves to calculate AUC and C-index metrics to assess their discriminatory power. Clinical decision curves were constructed to determine the clinical utility of the models.

To evaluate the predictive capabilities of the scoring models across diverse patient cohorts, subgroup analyses were executed within pre-specified subgroups: age (≥65/<65 years), sex (male/female), HBV infection (yes/no), refractory ascites (yes/no) and hepatocellular carcinoma (yes/no). The C-index for each model was computed within these subgroups to discern any significant disparities. The surv_cutpoint function from the R survminer package was employed to ascertain the optimal cutoff values for predicting outcomes with each score. Utilizing these cutoffs, patients were stratified into high or low-risk categories. Kaplan-Meier curves were plotted for these categories, followed by log-rank tests for comparison. Cox regression analysis was conducted to validate the HR. All statistical analyses were performed using R software (version 4.3.0). The models’ calibration was illustrated using the ‘QHScrnomo’ package. For ROC curves, the ‘riskRegression’, ‘ggprism’ and ‘ggplot2’ packages were used, while ‘ggDCA’ was applied for clinical decision curve analysis and ‘survminer’ for survival analysis. All tests were two-tailed, and a *p* value less than .05 was deemed to indicate statistical significance.

## Results

### Baseline characteristics

**Figure 1.**
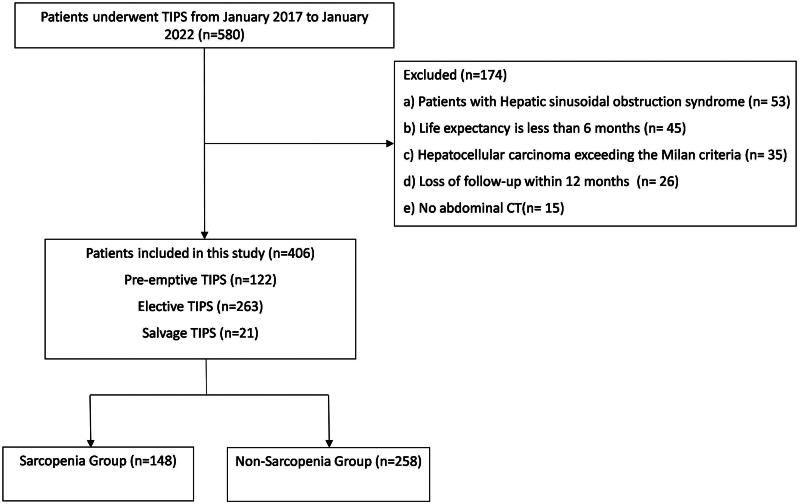
Flowchart of patient selection.

A total of 580 patients underwent TIPS during the study period. Among them, 174 patients who did not meet this study criteria were excluded, leaving a final cohort of 406 patients (Figure 1). There were 293 males and 113 females with a median age of 54 years (range: 47–62 years). Hepatitis B virus was the most prevalent cause of cirrhosis, affecting 56.16% of the study patients. Among the cohort, 12.81% (52 patients) had HCC within the Milan criteria. Patients with concurrent ascites were 69.46% (282 cases); 16.50% (67 cases) of the patients had concurrent diabetes mellitus. The median value of total bilirubin was 21.10 µmol/L (range: 13.95 µmol/L to 31.18 µmol/L). The median value of albumin was 32.60 g/L (range: 29.22 g/L to 36.10 g/L). The median value of the international normalized ratio was 1.24 (range: 1.12 to 1.40). The median value of creatinine was 63.00 µmol/L (range: 52.00 µmol/L to 78.00 µmol/L). The patients underwent TIPS to prevent rebleeding from oesophagogastric variceal bleeding [67.70% (275 patients)], refractory ascites [22.9% (93 patients)] and both [9.40% (38 patients)]. The median Child-Pugh score for the cohort was 7.0, with a range of 6–9, and the median MELD score was 10, within a range of 7–12. The median CLIFC-AD was 52.23 within a range of 42.53–64.17, and the median FIPS score was −1.06, ranging from −1.64 to −0.48. The median TPMT/H ratio was 11.32 mm/m (range: 9.06–14.12 mm/m) (Supplementary Table 1).

### Model evaluation

**Figure 2.**
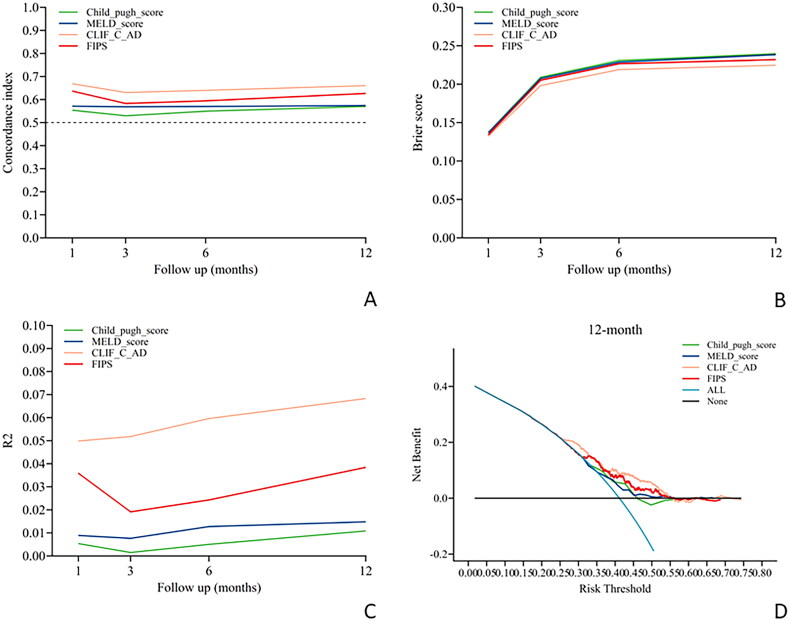
Performance of the four scores in predicting overt HE after TIPS procedure over time. (A) Time-dependent C-index evaluating discrimination. (B) Time-dependent brier score evaluating calibration. (C) Time-dependent R^2^ evaluating overall performance. (D) Decision curve analysis evaluating net benefit at 12 months after TIPS placement. CLIFC-AD: CLIF consortium acute decompensation; FIPS: Freiburg index of post-TIPS survival; MELD: model for end-stage liver disease; TIPS: transjugular intrahepatic portosystemic shunt.

**Figure 3.**
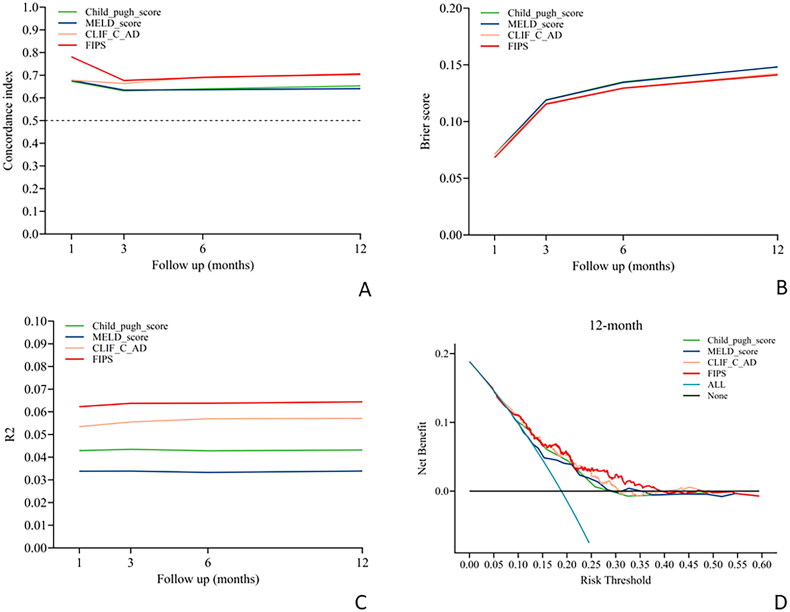
Performance of the four scores in predicting severe HE after TIPS procedure over time. (A) Time-dependent C-index evaluating discrimination. (B) Time-dependent Brier score evaluating calibration. (C) Time-dependent R2 evaluating overall performance. (D) Decision curve analysis evaluating net benefit at 12 months after TIPS placement. CLIFC-AD: CLIF consortium acute decompensation; FIPS: Freiburg index of post-TIPS survival; MELD: model for end-stage liver disease; TIPS: transjugular intrahepatic portosystemic shunt.

The evaluation of the four scoring models for their capacity to predict the risk of overt HE following the TIPS procedure revealed suboptimal performance across all time points post-TIPS. All models showed limited predictive ability at 1, 3, 6 and 12 months after TIPS, with a C-index below 0.7, reflecting poor performance (Table 1 and Figure 2A–D). Notably, the FIPS model exhibited superior predictive capability for the risk of severe HE at 1- and 12-months post-TIPS, achieving C-index values of 0.781 and 0.705, respectively (Table 1 and Figure 3A). The CLIFC AD model also demonstrated significant predictive power for severe HE at 12 months post-TIPS, with a C-index of 0.701 (Table 1 and Figure 3A). In contrast, the Child-Pugh and MELD models consistently showed lower predictive accuracy for severe HE at all examined time points post-TIPS, with C-index values remaining below the 0.7 threshold (Table 1 and Figure 3A).

**Table 1.** Performance of different scores in the prediction of post-TIPS HE at 1, 3, 6 and 12 months.

Score	C-index		Brier score		R^2^	
	Overt HE	Severe HE	Overt HE	Severe HE	Overt HE	Severe HE
1 month						
Child_Pugh	0.554	0.673	0.137	0.071	0.005	0.043
MELD	0.571	0.678	0.137	0.071	0.009	0.034
CLIFC-AD	0.669	0.679	0.132	0.071	0.050	0.053
*FIPS*	*0.637*	*0.781*	*0.134*	*0.068*	*0.036*	*0.062*
3 months						
Child_Pugh	0.530	0.631	0.209	0.119	0.002	0.043
MELD	0.568	0.635	0.207	0.119	0.008	0.034
CLIFC-AD	0.631	0.663	0.198	0.116	0.052	0.056
FIPS	0.583	0.677	0.205	0.115	0.019	0.064
6 months						
Child_Pugh	0.549	0.640	0.231	0.135	0.005	0.043
MELD	0.570	0.636	0.229	0.134	0.013	0.033
CLIFC-AD	0.640	0.693	0.219	0.130	0.060	0.057
FIPS	0.594	0.690	0.227	0.129	0.024	0.064
12 months						
Child_Pugh	0.570	0.653	0.240	0.148	0.011	0.043
MELD	0.574	0.641	0.238	0.148	0.015	0.034
*CLIFC-AD*	*0.660*	*0.701*	*0.225*	*0.142*	*0.068*	*0.057*
*FIPS*	*0.626*	*0.705*	*0.232*	*0.141*	*0.038*	*0.064*

*Notes:* Concordance (C)-index represents the measure of discrimination whereby values closer to 1 indicate better discriminative ability. The Brier Score is a metric quantifying the accuracy of probabilistic predictions, where values closer to 0 indicate higher predictive precision. R² measures the goodness-of-fit in regression models, with values closer to 1 denoting stronger explanatory power of the model. CLIFC-AD: CLIF consortium acute decompensation; FIPS: Freiburg index of post-TIPS survival; MELD: model for end-stage liver disease; TIPS: transjugular intrahepatic portosystemic shunt.

In predicting severe HE post-TIPS, the FIPS score consistently surpassed the performance of the other three models. It demonstrated the most favourable Brier scores at all critical time points, indicating superior calibration (Table 1 and Figure 3B). Additionally, the FIPS score attained the highest R^2^ values at each significant post-operative time point, signifying more precise predictions (Table 1 and Figure 3C). Decision curve analysis further indicated that the FIPS scoring model provided a greater net benefit across various threshold probabilities, highlighting its enhanced clinical utility compared to the other models (Figure 3D).

### Subgroup analysis

The four scoring models demonstrated lower predictive performance for post-TIPS overt HE six months after the procedure across different patient subgroups with C-index scores below 0.7 (Supplementary Figure 1). Notably, the FIPS scoring model showed a relatively higher predictive performance for severe HE six months post-TIPS in patients with HBV infection, achieving a C-index of 0.716 (Supplementary Figure 2D). In contrast, the other scoring models had C-index scores below 0.70 for predicting severe HE in the same subgroups (Supplementary Figure 2).

### Sarcopenia subgroup model evaluation

**Figure 4.**
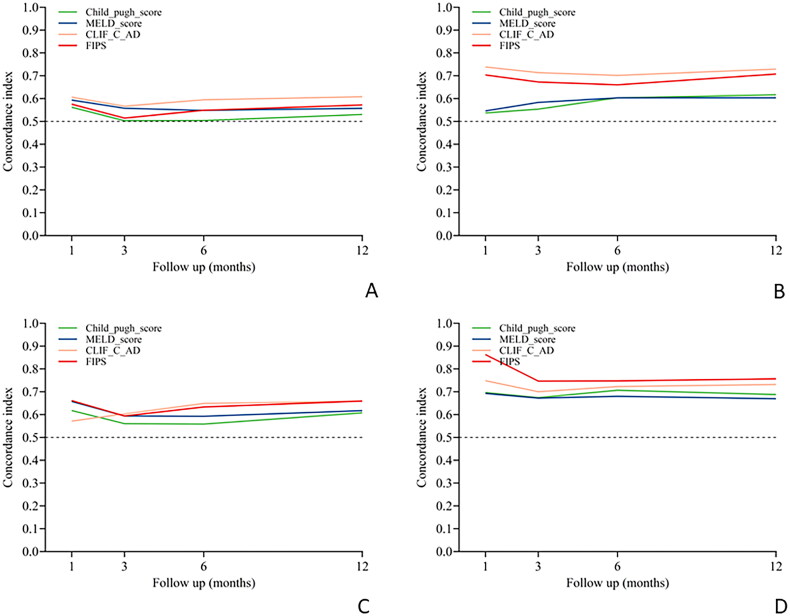
Performance of the four scores in predicting HE after TIPS procedure over time in the sarcopenic (Sa) and non-sarcopenic (non-Sa) patients. (A) Time-dependent C-index evaluating discrimination of overt HE in the non-Sa group. (B) Time-dependent C-index evaluating discrimination of overt HE in the Sa group. (C) Time-dependent C-index evaluating discrimination of severe HE in the non-Sa group. (D) Time-dependent C-index evaluating discrimination of severe HE in the Sa group. CLIFC-AD: CLIF consortium acute decompensation; FIPS: Freiburg index of post-TIPS survival; MELD: model for end-stage liver disease; TIPS: transjugular intrahepatic portosystemic shunt.

The four scoring models demonstrated low predictive performance for the risk of overt and severe HE at 1, 3, 6 and 12 months post-TIPS in patients without sarcopenia, with all C-index values below 0.7 (Table 2, Figure 4A,C).

**Table 2.** Performance of different scores in the prediction of post-TIPS HE at 1, 3, 6 and 12 months in sarcopenic and non-sarcopenic patients.

Score	C-index			
	Overt HE		Severe HE	
	Non-Sa group	Sa group	Non-Sa group	Sa group
1 month				
Child_Pugh	0.562	0.537	0.618	0.697
MELD	0.593	0.546	0.657	0.692
CLIFC-AD	0.606	0.738	0.571	0.748
FIPS	0.575	0.703	0.661	0.863
3 months				
Child_Pugh	0.503	0.554	0.560	0.674
MELD	0.557	0.583	0.594	0.672
CLIFC-AD	0.567	0.714	0.603	0.700
FIPS	0.515	0.673	0.594	0.747
6 months				
Child_Pugh	0.504	0.603	0.558	0.707
MELD	0.548	0.603	0.593	0.680
CLIFC-AD	0.595	0.702	0.649	0.723
FIPS	0.549	0.660	0.633	0.747
12 months				
Child_pugh	0.530	0.617	0.607	0.688
MELD	0.557	0.603	0.617	0.669
CLIFC-AD	0.608	0.729	0.659	0.732
FIPS	0.572	0.707	0.659	0.757

*Notes:* Concordance (C)-index represents the measure of discrimination whereby values closer to 1 indicate better discriminative ability. CLIF-C AD: CLIF consortium acute decompensation; FIPS: Freiburg index of post TIPS survival; MELD: model for end-stage liver disease; Sa: sarcopenia; TIPS: transjugular intrahepatic portosystemic shunt.

In contrast, for sarcopenic patients, the FIPS scoring model had good predictive performance for the risk of overt HE at 1 month and 12 months post-TIPS, with C-index values of 0.703 and 0.707, respectively (Table 2, Figure 4B). Similarly, the CLIFC-AD model showed good predictive ability for the risk of overt HE at 1, 3, 6 and 12 months in sarcopenic patients, with C-index values of 0.738, 0.714, 0.702 and 0.729, respectively (Table 2, Figure 4B).

For severe HE, the FIPS model exhibited the best predictive performance at 1, 3, 6 and 12 months post-TIPS in sarcopenic patients, with C-index values of 0.863, 0.747, 0.747 and 0.757, respectively (Table 2, Figure 4D), whereas the CLIFC-AD model also showed commendable predictive ability for severe HE at these time points, with C-index values of 0.748, 0.700, 0.723 and 0.732, respectively (Table 2, Figure 4D).

### Risk stratification

**Figure 5.**
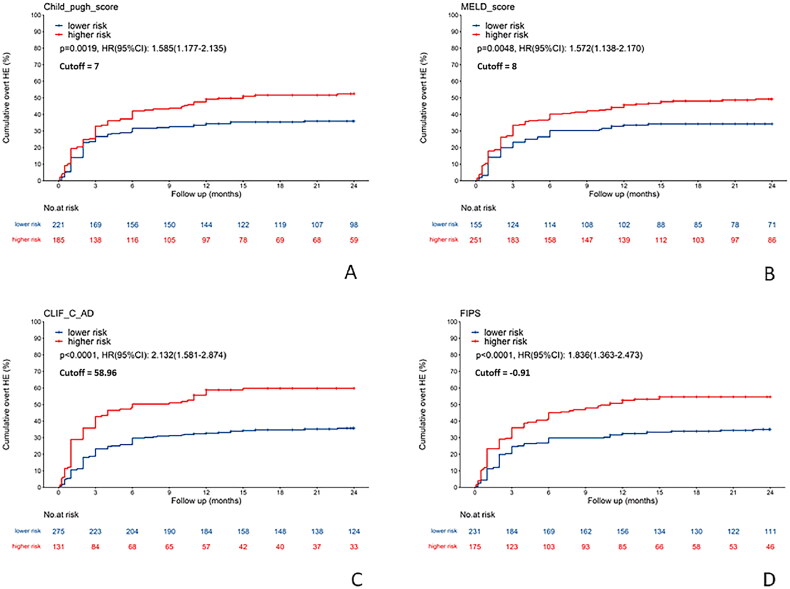
Comparison of the cumulative incidence of overt hepatic encephalopathy after TIPS procedure between high-risk and low-risk groups determined by four scoring models. (A) Child-Pugh score (cutoff: 7). (B) MELD score (cutoff: 8). (C) CLIFC-AD score (cutoff: 58.96). (D) FIPS score (cutoff: −0.91). CLIFC-AD: CLIF consortium acute decompensation; FIPS: Freiburg index of post-TIPS survival; MELD: model for end-stage liver disease; TIPS: transjugular intrahepatic portosystemic shunt.

The study cohort was categorized into low-risk and high-risk groups for overt HE post-TIPS based on optimal cutoff values determined by the surv_cutpoint function. The high-risk groups, as delineated by the four scoring models, demonstrated a significantly higher cumulative incidence of overt HE compared to their low-risk counterparts. Specifically, the Child-Pugh model indicated an HR of 1.585 (95% CI: 1.177–2.135), the MELD model presented an HR of 1.572 (95% CI: 1.138–2.170), the CLIFC-AD model showed an HR of 2.132 (95% CI: 1.581–2.874), and the FIPS model had an HR of 1.836 (95% CI: 1.363–2.473) (Figure 5).

**Figure 6.**
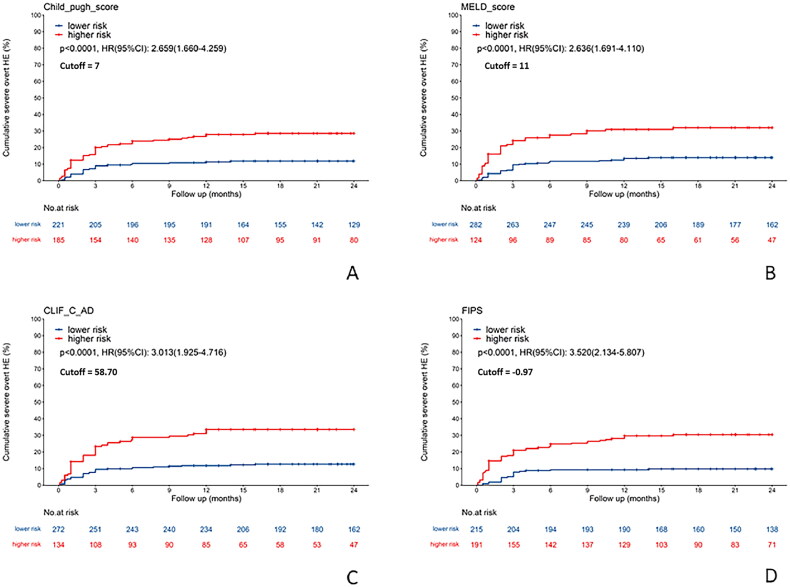
Comparison of the cumulative incidence of severe hepatic encephalopathy after TIPS between high-risk and low-risk groups determined by four scoring models. (A) Child-Pugh score (cutoff: 7). (B) MELD score (cutoff: 11). (C) CLIFC-AD score (cutoff: 58.70). (D) FIPS score (cutoff: −0.97). CLIFC-AD: CLIF consortium acute decompensation; FIPS: Freiburg index of post-TIPS survival; MELD: model for end-stage liver disease; TIPS: transjugular intrahepatic portosystemic shunt.

Additionally, the high-risk groups were linked with a significantly increased cumulative incidence of severe HE following TIPS. The Child-Pugh model exhibited an HR of 2.659 (95% CI: 1.660–4.259), the MELD model displayed an HR of 2.636 (95% CI: 1.691–4.110), the CLIFC-AD model reported an HR of 3.013 (95% CI: 1.925–4.716), and the FIPS model showed an HR of 3.520 (95% CI: 2.134–5.807), with all *p* values being less than .001 (Figure 6).

## Discussion

This study evaluated the Child-Pugh, MELD, CLIFC-AD and FIPS scoring models in predicting the risk of HE after TIPS. The findings revealed that all four models had limited efficacy in forecasting the risk of overt HE at 1, 3, 6 and 12 months post-TIPS, with C-index values all below 0.7. However, the FIPS model emerged as the most effective for predicting severe HE at 1 and 12 months, with C-indices of 0.781 and 0.705, respectively. The CLIF-C AD model also demonstrated notable predictive capability for severe HE at 12 months post-TIPS, with a C-index of 0.701. These results highlight the significant clinical potential of the FIPS and CLIFC AD models for predicting severe HE following TIPS.

The traditional Child-Pugh score includes subjective variables such as ascites and HE, which may lead to assessment variability among evaluators. As a categorical scoring system that assigns equal weight to all variables and spans a range of 0–15 points, the Child-Pugh score may not effectively predict patient outcomes after TIPS. In contrast, the MELD score, which incorporates objective measures like serum creatinine, bilirubin and the international normalized ratio, is a predictive model for mortality risk and has been favoured in clinical settings for evaluating TIPS procedures. Nonetheless, it lacks the inclusion of serum albumin levels, an independent predictor of post-TIPS HE. Serum albumin reflects the liver’s synthetic function and the patient’s nutritional status, underscoring its importance in the prognosis of HE after TIPS [18,19]. Advanced age further compounds the risk of HE due to diminished organ function and detoxification capabilities [4,20,21]. Hyponatremia, frequently observed in decompensated cirrhosis, can exacerbate cerebral edoema and elevate the risk of HE [22,23]. Additionally, bacterial infections, indicated by increased white blood cell (WBC) counts [24], may precipitate HE, as there is a strong association between systemic inflammation and severe HE (Grade 3/4 HE) [25]. The FIPS score integrates age and serum albumin, whereas the CLIFC AD score incorporates age, WBC and serum sodium into its predictive model. This enhancement significantly improves the prediction of post-TIPS severe HE compared to the Child-Pugh and MELD scores, as illustrated by this study.

Our findings indicate that the four scoring models were somewhat limited in predicting overt HE at various post-TIPS intervals, with all C-indexes below 0.7. However, these models showed improved effectiveness in forecasting the risk of severe HE at each assessed post-TIPS time point, as detailed in Table 1. This advancement implies that the grading of HE substantially impacts model accuracy. The diagnosis of Grade II HE poses particular challenges due to its subjective nature and variability. It is essential to differentiate between Grade II HE and Grade III/IV HE, as patients in the latter category present with more severe symptoms, necessitate more aggressive management and have a differing prognosis [26–28]. Specifically, patients with severe HE often require hospitalization for specialized treatments like ornithine aspartate, lactulose and rifaximin [7], and their condition may primarily be due to compromised hepatic functional reserve [26,27]. Meanwhile, patients with Grade II HE generally have better hepatic functional reserve. Consequently, this could be why the four scoring models, designed to reflect hepatic reserve, are more accurate in predicting severe HE.

When predicting the risk of post-TIPS overt HE, the four scoring models demonstrated uniform performance across various subgroups (age, gender, HBV infection, refractory ascites and HCC). However, the sarcopenia subgroup yielded intriguing findings. Sarcopenia, characterized by diminished muscle mass, is widely prevalent in cirrhotic patients, with rates ranging from 40 to 70% [29,30].

Notably, sarcopenia has been linked with an elevated risk of HE, as evident by a meta-analysis of six studies encompassing 1795 patients, which revealed a strong positive association (OR = 2.74, 95% CI= 1.87–4.01) [31]. Furthermore, a focused study found that cirrhotic patients with sarcopenia were 31.3 times more likely to develop HE after TIPS compared to non-sarcopenic patients [32]. This increased risk was potentially due to the role of skeletal muscles in regulating ammonia levels. After TIPS, patients often experience hyperammonemia, which can worsen with sarcopenia, while the deleterious effects of high ammonia levels may further deteriorate muscle mass, creating a detrimental feedback loop.

This study stratified patients based on the presence of sarcopenia prior to undergoing TIPS. The four scoring models were not ideal for predicting overt and severe HE post-TIPS in non-sarcopenic patients, with all C-index values being below 0.70. However, there was a notable improvement in the prediction of overt HE in sarcopenic patients, with C-index values ranging from 0.537 to 0.738. This finding suggests that in addition to hepatic functional reserve, sarcopenia may also be an important cause of overt HE. The prediction was even more accurate in predicting post-TIPS severe HE in sarcopenic patients, with C-index values between 0.672 and 0.863. The FIPS and CLIFC-AD models stood out, especially for sarcopenic patients, showing the most significant predictive improvement for both post-TIPS overt and severe HE. The FIPS model demonstrated superior predictive ability for severe HE, with C-index values at 1, 3, 6 and 12 months post-TIPS of 0.863, 0.747, 0.747 and 0.757, respectively. Similarly, the CLIFC-AD model had good predictive capability, with respective C-index values of 0.748, 0.700, 0.723 and 0.732. These findings indicate that both the FIPS and CLIFC-AD models could be reliably used in clinical practice to predict the risk of severe HE after TIPS in patients with sarcopenia. The significant improvement in the predictive ability of the scoring models for post-TIPS HE in sarcopenic patients underscores the importance of considering sarcopenia in the mechanism of HE development post-TIPS. Furthermore, this study paves the way for future research to explore a combined model incorporating FIPS and CLIFC-AD scores with the sarcopenia variable to potentially enhance predictive accuracy for HE risk after TIPS.

Patients categorized as high-risk by the four clinical scoring models demonstrated significantly higher cumulative incidences of overt and severe HE following TIPS. Notably, the FIPS exhibited the highest risk ratio for severe HE post-TIPS (HR = 3.520, 95% CI: 2.134–5.807), while the CLIFC-AD model was most predictive of overt HE (HR = 2.132, 95% CI: 1.581–2.874). These findings indicate that the FIPS model is superior in identifying the risk of severe HE, and the CLIFC-AD model excels in predicting overt HE after TIPS.

A comparison of the predictive efficacy of scoring systems for HE after TIPS has rarely been performed. Wang et al. retrospectively analysed the clinical data of 195 patients with liver cirrhosis and portal hypertension who underwent TIPS [33]. They confirmed that the predictive efficacy of indocyanine green retention rate at 15 min (ICG-R15) for post-TIPS HE (AUC = 0.664) was superior to that of the Child-Pugh score (AUC = 0.596) and the MELD score (AUC = 0.641). An ICG-R15 > 30% could effectively distinguish high-risk populations (HE incidence: 28.5% vs 13%). The present study included the clinical data of 406 patients with liver cirrhosis and portal hypertension who underwent TIPS. It systematically compared the predictive efficacy of novel scoring systems such as FIPS and CLIFC-AD) with traditional scoring systems (Child-Pugh score and MELD score) for post-TIPS HE. Moreover, it integrated sarcopenia stratification analysis for the first time. The results showed that the predictive efficacy of the Child-Pugh and MELD scores for OHE was similar to that reported by Wang et al. [33]. However, novel scoring systems such as FIPS and CLIFC-AD demonstrated good predictive efficacy for severe HE. In particular, the FIPS score had the optimal predictive efficacy for severe HE one month after TIPS (C-index = 0.781) and it significantly increased to 0.863 in the sarcopenia subgroup, with a hazard ratio as high as 3.52. This highlights the regulatory role of muscle status in HE risk stratification and contributes to optimizing the individualized management of high-risk patients.

According to the data in Figure 6, the HR of the FIPS scoring model is 3.520 (95% CI: 2.134–5.807), significantly higher than that of other models. This indicates that the FIPS model has the optimal ability to distinguish between high- and low-risk patients. The risk threshold of the model is −0.97, suggesting that patients with a FIPS score greater or equal to −0.97 (high-risk group) have a 3.5-fold higher cumulative risk of severe HE within 12 months after TIPS compared to the low-risk group. Clinically, preventive interventions should be preferentially implemented for such patients, including preoperative nutritional intervention, the use of small-diameter stents during the operation and post-operative oral administration of rifaximin to inhibit intestinal bacteria, thereby reducing the risk of HE after TIPS.

This study has several strengths. Firstly, it utilizes a large-sample TIPS cohort, thoroughly analysing and comparing the predictive values of four scoring models for the risk of overt and severe HE post-TIPS. Each participant underwent TIPS with a covered stent, minimizing the potential impact of bare stents on outcomes and ensuring adherence to the follow-up plan, which enhanced the data’s integrity and reliability. Secondly, the HE predictive model is based on the occurrence of overt and severe HE within specific time frames (1, 3, 6 and 12 months post-TIPS). Thirdly, the FIPS score, initially developed from a modern European cohort and lacking external validation, has been validated through this study to predict the risk of HE post-TIPS in a Chinese population.

However, the study also acknowledges certain limitations. This research is a single-centre retrospective study, which indicates that the variable procedural complexities of TIPS and the diverse clinical profiles of patients at different institutions call for external validation through multicentre, high-quality, large-sample longitudinal studies. Furthermore, this study cohort predominantly comprised patients with hepatitis B virus-related cirrhosis and the primary indications for TIPS were oesophagogastric variceal bleeding or refractory ascites. This may constrain the generalizability of our findings to populations with alternative aetiologies (e.g. alcohol-associated cirrhosis, metabolic dysfunction-associated steatotic liver disease [MASLD]) or TIPS indications (e.g. Budd-Chiari syndrome and hepatic hydrothorax). Consequently, future studies should prioritize external validation in cirrhotic cohorts encompassing diverse aetiologies (alcohol-related, MASLD and autoimmune hepatitis) and TIPS indications (such as Budd-Chiari syndrome and hepatic hydrothorax).

Moreover, the manuscript presents the evaluation of sarcopenia by measuring TPMT using CT imaging. However, this method may not comprehensively capture the complexity of sarcopenia, especially considering other factors such as muscle function and fat infiltration (myosteatosis). Several tools are available for the objective assessment of sarcopenia [34,35]. Thus, the significant findings in the sarcopenic subgroup observed in this study may undergo further validation and replication using additional sarcopenia assessment tools to improve the robustness and generalizability of the results.

## Conclusions

In summary, the study found that both the FIPS and CLIF-C AD scoring models possess strong predictive capabilities for assessing the risk of severe HE following TIPS, particularly among sarcopenic patients. These models proved effective in risk stratification for severe and overt HE, supporting their use in clinical practice. These findings not only provide more accurate tools for predicting post-operative HE but also offer a theoretical basis for the development of personalized treatment strategies, particularly for patients with sarcopenia.

## Supplementary Material

Supplemental Material

## Data Availability

The datasets generated and analysed during the present study are available from the corresponding author on reasonable request.
