# Supplementary material for: Comparing efficacy of different scoring models to predict hepatic encephalopathy after TIPS in cirrhotic patients
Source: Ann Med. 2025 Jun 6;57(1):2514082. doi: 10.1080/07853890.2025.2514082 (PMC12147511; doi:10.1080/07853890.2025.2514082)
Supplement: Supplemental Material [file IANN_A_2514082_SM8868.zip › suppl_data/Supplementary figure legends_Apr15.docx]

**Supplementary Figure 1.** Forest plots demonstrating the discriminative ability of the four scoring systems for overt HE in prespecified subgroups. (A) Child- -Pugh score. (B) MELD score. (C) CLIFC-AD score. (D) FIPS score. Concordance (c)-index ranges from 0 to 1, and a higher value indicates better discrimination. The prognostic performance of each score did not vary significantly based on these subgroups. CLIFC-AD = CLIF consortium acute decompensation, FIPS = Freiburg index of post-TIPS survival, MELD = model for end-stage liver disease, TIPS = transjugular intrahepatic portosystemic shunt.

**Supplementary Figure 2.** Forest plots demonstrating the discriminative ability of the four scoring systems for severe HE in prespecified subgroups. (A) Child- -Pugh score. (B) MELD score. (C) CLIFC-AD score. (D) FIPS score. Concordance (c)-index ranges from 0 to 1, and a higher value indicates better discrimination. The prognostic performance of each score did not vary significantly based on these subgroups. CLIFC-AD = CLIF consortium acute decompensation, FIPS = Freiburg index of post-TIPS survival, MELD = model for end-stage liver disease, TIPS = transjugular intrahepatic portosystemic shunt.
