# Supplementary material for: Comparing efficacy of different scoring models to predict hepatic encephalopathy after TIPS in cirrhotic patients
Source: Ann Med. 2025 Jun 6;57(1):2514082. doi: 10.1080/07853890.2025.2514082 (PMC12147511; doi:10.1080/07853890.2025.2514082)
Supplement: Supplemental Material [file IANN_A_2514082_SM8868.zip › suppl_data/Supplementary_Table_1_Apr15 - clean.docx]

**Supplementary Table 1 Baseline characteristics comparison between the overall study population and the sarcopenia/non-sarcopenia groups**

| **Variable** | **Overall cohort** | **Non-sarcopenia group** | **Sarcopenia group** | **P value** |
| --- | --- | --- | --- | --- |
|  | （N=406） | （N=258） | （N=148） |  |
| **Age(years)** | 54.00 [47.00, 62.00] | 53.00 [47.00, 60.00] | 55.00 [49.00, 64.25] | 0.005 |
| **Age_score** |  |  |  | 0.162 |
| **< 65 years** | 321 (79.06%) | 210 (81.40%) | 111 (75.00%) |  |
| **≥ 65 years** | 85 (20.94%) | 48 (18.60%) | 37 (25.00%) |  |
| **Gender** |  |  |  | 0.147 |
| **Male** | 293 (72.17%) | 193 (74.81%) | 100 (67.57%) |  |
| **Female** | 113 (27.83%) | 65 (25.19%) | 48 (32.43%) |  |
| **Height (m)** | 1.66 [1.60,1.70] | 1.67 [1.60,1.71] | 1.66 [1.60, 1.70] | 0.130 |
| **BMI (kg/m^2^)** | 22.74 [20.76, 24.22] | 22.86 [21.38, 24.66] | 22.17 [20.44, 23.53] | 0.001 |
| **Aetiology** |  |  |  | 0.218 |
| **No** | 237 (58.37%) | 157 (60.85%) | 80 (54.05%) |  |
| **Yes** | 169 (41.63%) | 101 (39.15%) | 68 (45.95%) |  |
| **HBV** |  |  |  | 0.338 |
| **No** | 178 (43.84%) | 108 (41.86%) | 70 (47.30%) |  |
| **Yes** | 228 (56.16%) | 150 (58.14%) | 78 (52.70%) |  |
| **Diabetes** |  |  |  | 0.417 |
| **No** | 339 (83.50%) | 212 (82.17%) | 127 (85.81%) |  |
| **Yes** | 67 (16.50%) | 46 (17.83%) | 21 (14.19%) |  |
| **Hypertension** |  |  |  | 0.719 |
| **No** | 361 (88.92%) | 231 (89.53%) | 130 (87.84%) |  |
| **Yes** | 45 (11.08%) | 27 (10.47%) | 18 (12.16%) |  |
| **Ascites** |  |  |  | 0.134 |
| **No** | 124 (30.54%) | 86 (33.33%) | 38 (25.68%) |  |
| **Yes** | 282 (69.46%) | 172 (66.67%) | 110 (74.32%) |  |
| **HCC** |  |  |  | 0.634 |
| **No** | 354 (87.19%) | 227 (87.98%) | 127 (85.81%) |  |
| **Yes** | 52 (12.81%) | 31 (12.02%) | 21 (14.19%) |  |
| **Pre-TIPS OHE** |  |  |  | 0.149 |
| **No** | 389(95.81%) | 250(96.90%) | 139(93.92%) |  |
| **Yes** | 17(4.19%) | 8(3.10%) | 9(6.08%) |  |
| **WBC (×10^9^/L)** | 3.51 [2.37, 5.46] | 3.80 [2.40, 5.52] | 3.20 [2.34, 5.13] | 0.214 |
| **RBC (×10^12^/L)** | 2.91 [2.45, 3.48] | 2.97 [2.48, 3.61] | 2.86 [2.39, 3.28] | 0.007 |
| **Hemoglobin (g/L)** | 80.00 [65.47,100.00] | 81.50 [66.00,100.75] | 77.00 [63.00, 93.00] | 0.028 |
| **Platelets (×10^9^/L)** | 67.00 [45.00,105.75] | 67.50 [46.00,109.00] | 64.00 [43.00, 96.50] | 0.190 |
| **ALT (U/L)** | 22.00 [15.00, 33.00] | 23.00 [16.00, 35.45] | 21.00 [14.75, 29.00] | 0.024 |
| **AST (U/L)** | 30.00 [22.00, 42.00] | 30.00 [21.70, 43.00] | 30.50 [23.75, 41.00] | 0.839 |
| **TBIL (umol/L)** | 21.10 [13.95, 31.18] | 20.65 [14.40, 27.75] | 21.55 [13.38, 34.47] | 0.755 |
| **DBIL (umol/L)** | 9.40 [6.12, 15.60] | 9.25 [6.12, 14.68] | 9.90 [6.18, 17.12] | 0.505 |
| **IBIL (umol/L)** | 10.25 [6.93, 15.38] | 10.15 [7.23, 15.05] | 10.40 [6.53, 16.52] | 0.813 |
| **Albumin (g/L)** | 32.60 [29.22,36.10] | 32.80 [29.45,36.82] | 32.25 [28.37,35.60] | 0.129 |
| **Prealbumin (g/L)** | 92.00 [66.00, 119.00] | 95.50 [69.00, 122.00] | 87.50 [62.75, 118.00] | 0.082 |
| **Globulin (g/L)** | 27.45 [23.00, 32.38] | 27.00 [22.70, 31.50] | 28.05 [23.75, 33.73] | 0.047 |
| **Lymphocyte (×10^9^/L)** | 0.70 [0.46, 1.10] | 0.78 [0.49, 1.23] | 0.64 [0.42, 0.87] | <0.001 |
| **BUN (mmol/L)** | 5.86 [4.38, 8.09] | 5.77 [4.31, 7.82] | 6.03 [4.48, 8.31] | 0.290 |
| **Creatinine (umol/L)** | 63.00 [52.00, 78.00] | 63.00 [53.00, 76.00] | 64.00 [52.00, 80.00] | 0.603 |
| **K^+^(mmol/L)** | 3.91 [3.58, 4.20] | 3.88 [3.57, 4.16] | 3.93 [3.60, 4.27] | 0.461 |
| **Na^+^ (mmol/L)** | 139.00 [135.16, 141.29] | 139.14 [136.00, 141.71] | 138.51 [134.35, 141.00] | 0.033 |
| **Cl^+^ (mmol/L)** | 104.55 [100.12, 107.50] | 105.00 [101.53, 107.69] | 103.85 [97.85, 107.00] | 0.011 |
| **PT (s)** | 15.00 [13.50, 16.78] | 15.00 [13.43, 16.90] | 15.00 [13.57, 16.40] | 0.877 |
| **INR** | 1.24 [1.12, 1.40] | 1.25 [1.12, 1.43] | 1.24 [1.11, 1.40] | 0.786 |
| **APTT (s)** | 38.00 [32.73, 43.50] | 38.15 [32.85, 43.08] | 37.90 [32.68, 44.12] | 0.755 |
| **TT (s)** | 17.40 [16.00, 18.80] | 17.60 [16.20, 18.90] | 17.10 [15.57, 18.40] | 0.035 |
| **Ammonia (umol/L)** | 32.80 [21.95, 57.00] | 31.70 [19.60, 58.50] | 35.15 [24.05, 55.50] | 0.149 |
| **Child_pugh_score** | 7.00 [6.00, 9.00] | 7.00 [6.00, 8.00] | 8.00 [6.00, 9.00] | 0.054 |
| **MELD_score** | 10.00 [7.00, 12.00] | 10.00 [7.00, 12.00] | 10.00 [7.00, 12.00] | 0.951 |
| **CLIFC-AD _score** | 52.23[42.53, 64.17] | 50.48[41.24, 59.27] | 55.17[44.81, 69.42] | 0.008 |
| **FIPS_score** | -1.06 [-1.64, -0.48] | -1.10 [-1.65, -0.55] | -0.94 [-1.58, -0.29] | 0.120 |
| **TPMT/H (mm/m)** | 11.32 [9.06,14.12] | 13.25 [11.64,15.13] | 8.36 [6.88, 9.82] | <0.001 |
| **Post-TIPS PPG (mmHg)** | 14.0 [10.0,18.0] | 11.0 [9.0,17.0] | 15.0 [10.0,21.0] | <0.001 |
| **Post-TIPS OHE** |  |  |  | 0.003 |
| **No** | 231(56.90%) | 161(62.40%) | 70(47.30%) |  |
| **Yes** | 175(43.10%) | 97(37.60%) | 78(52.70%) |  |
| **Post-TIPS SHE** |  |  |  | 0.001 |
| **No** | 328(80.79%) | 221(85.66%) | 107(72.30%) |  |
| **Yes** | 78(19.21%) | 37(14.34%) | 41(27.70%) |  |

**ALT = alanine aminotransferase, APTT = activated partial thromboplastin time, AST = Aspartate aminotransferase, BMI = Body mass index, BUN = blood urea nitrogen, CLIFC-AD = CLIF consortium acute decompensation, DBIL = Direct Bilirubin, FIPS = Freiburg index of post-TIPS survival, HCC = hepatocellular carcinoma, IBIL = Indirect bilirubin, INR = international normalized ratio, MELD = model for end-stage liver disease, OHE = overt hepatic encephalopathy, PPG = portal pressure gradient, PT = prothrombin time, RBC = red blood cell count, SHE = severe hepatic encephalopathy, TBIL = total bilirubin, TIPS = transjugular intrahepatic portosystemic shunt, TPMT = transversal psoas muscle thickness, TT = thrombin time, WBC = white blood cell count.**
