# Supplementary figures and images for: Comparing efficacy of different scoring models to predict hepatic encephalopathy after TIPS in cirrhotic patients
Source: Ann Med. 2025 Jun 6;57(1):2514082. doi: 10.1080/07853890.2025.2514082 (PMC12147511; doi:10.1080/07853890.2025.2514082)

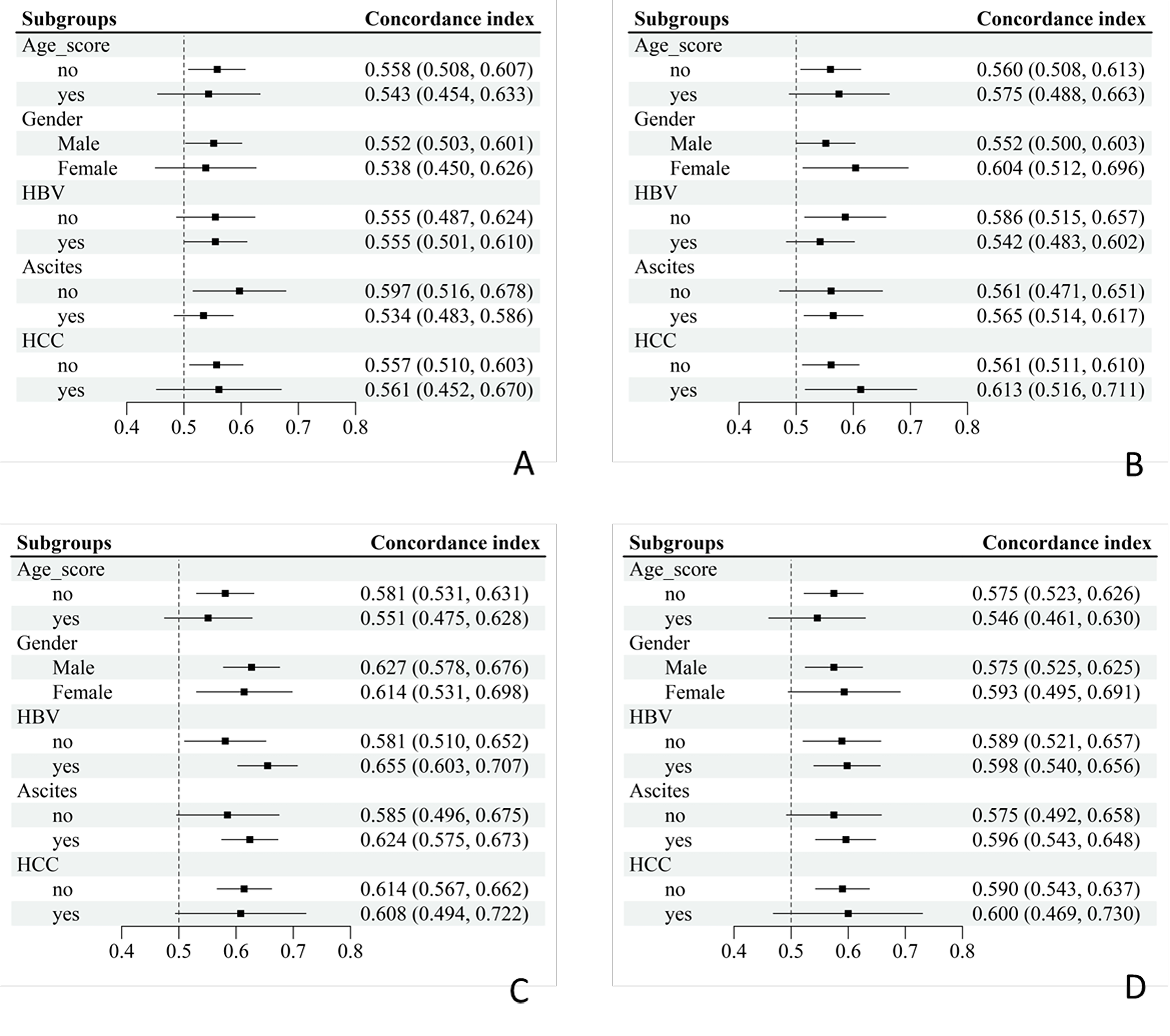

Supplement: Supplemental Material [file IANN_A_2514082_SM8868.zip › suppl_data/Supplementary Figure 1_Apr16.tif]

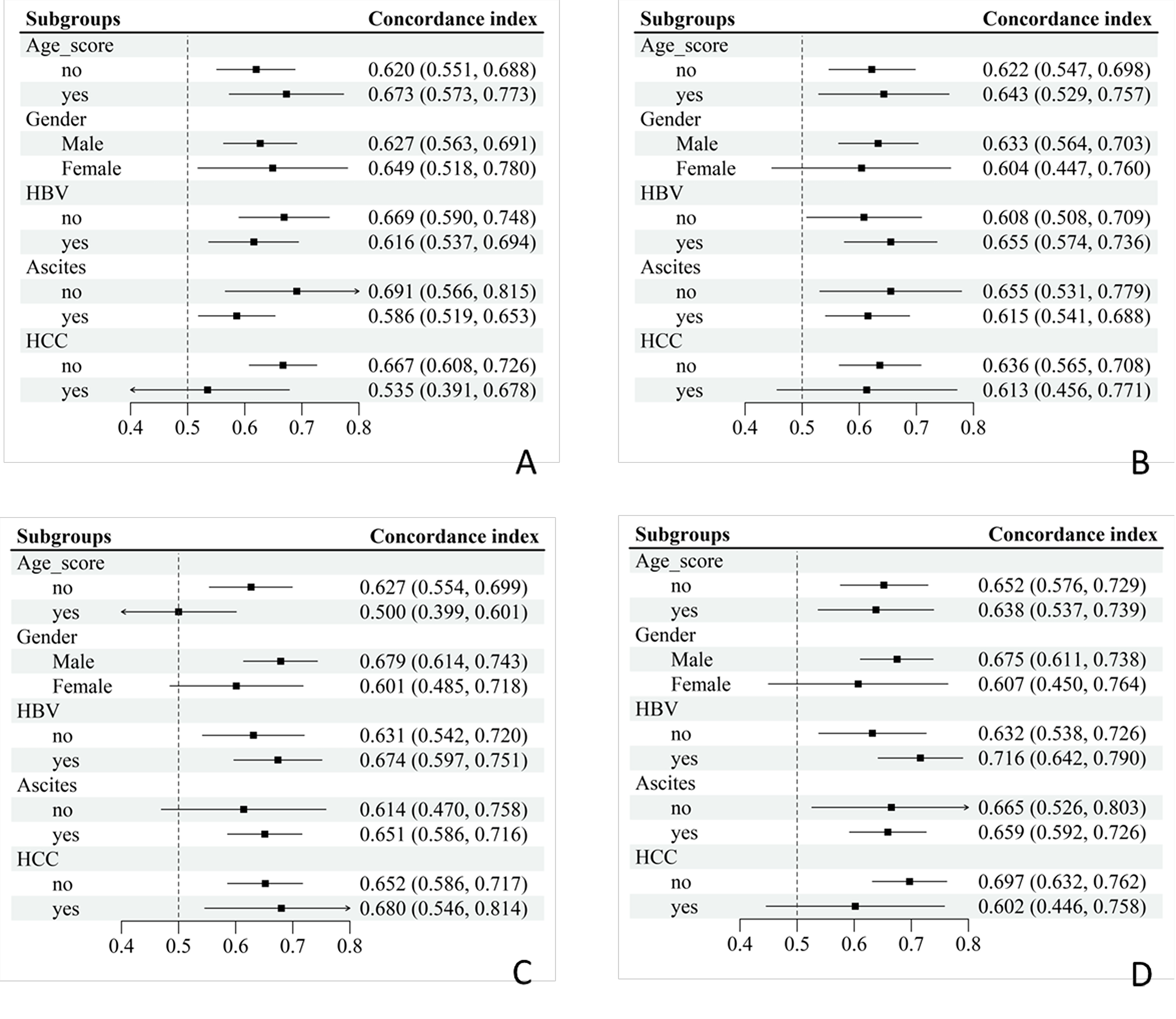

Supplement: Supplemental Material [file IANN_A_2514082_SM8868.zip › suppl_data/Supplementary Figure 2_Apr16.tif]
